# AC-265347 Inhibits Neuroblastoma Tumor Growth by Induction of Differentiation without Causing Hypocalcemia

**DOI:** 10.3390/ijms23084323

**Published:** 2022-04-13

**Authors:** Eliana Gonçalves-Alves, Marta Garcia, Carlos J. Rodríguez-Hernández, Soledad Gómez-González, Rupert C. Ecker, Mariona Suñol, Oscar Muñoz-Aznar, Angel M. Carcaboso, Jaume Mora, Cinzia Lavarino, Silvia Mateo-Lozano

**Affiliations:** 1 Developmental Tumor Biology Laboratory, Institut de Recerca Sant Joan de Déu, Hospital Sant Joan de Déu, 08950 Esplugues de Llobregat, Spain; eliana.alves@bio.ku.dk (E.G.-A.); marta.garcial@sjd.es (M.G.); carlos.rodriguez@sjd.es (C.J.R.-H.); soledad.gomezg@sjd.es (S.G.-G.); oscar.munoz@sjd.es (O.M.-A.); angel.montero@sjd.es (A.M.C.); jaume.mora@sjd.es (J.M.); cinzia.lavarino@sjd.es (C.L.); 2 Pediatric Cancer Center Barcelona, Hospital Sant Joan de Déu, 08950 Esplugues de Llobregat, Spain; 3 TissueGnostics GmbH, 1020 Vienna, Austria; rupert.ecker@tissuegnostics.com; 4 Department of Pathology, Hospital Sant Joan de Déu, 08950 Esplugues de Llobregat, Spain; mariona.sunol@sjd.es

**Keywords:** neuroblastoma, calcium-sensing receptor, calcimimetic, cinacalcet, AC-265347, biased signaling, differentiation, cancer-testis antigens

## Abstract

Neuroblastoma is the most common extracranial solid tumor of childhood, with heterogeneous clinical manifestations ranging from spontaneous regression to aggressive metastatic disease. The calcium-sensing receptor (CaSR) is a G protein-coupled receptor (GPCR) that senses plasmatic fluctuation in the extracellular concentration of calcium and plays a key role in maintaining calcium homeostasis. We have previously reported that this receptor exhibits tumor suppressor properties in neuroblastoma. The activation of CaSR with cinacalcet, a positive allosteric modulator of CaSR, reduces neuroblastoma tumor growth by promoting differentiation, endoplasmic reticulum (ER) stress and apoptosis. However, cinacalcet treatment results in unmanageable hypocalcemia in patients. Based on the bias signaling shown by calcimimetics, we aimed to identify a new drug that might exert tumor-growth inhibition similar to cinacalcet, without affecting plasma calcium levels. We identified a structurally different calcimimetic, AC-265347, as a promising therapeutic agent for neuroblastoma, since it reduced tumor growth by induction of differentiation, without affecting plasma calcium levels. Microarray analysis suggested biased allosteric modulation of the CaSR signaling by AC-265347 and cinacalcet towards distinct intracellular pathways. No upregulation of genes involved in calcium signaling and ER stress were observed in patient-derived xenografts (PDX) models exposed to AC-265347. Moreover, the most significant upregulated biological pathways promoted by AC-265347 were linked to RHO GTPases signaling. AC-265347 upregulated cancer testis antigens (CTAs), providing new opportunities for CTA-based immunotherapies. Taken together, this study highlights the importance of the biased allosteric modulation when targeting GPCRs in cancer. More importantly, the capacity of AC-265347 to promote differentiation of malignant neuroblastoma cells provides new opportunities, alone or in combination with other drugs, to treat high-risk neuroblastoma patients.

## 1. Introduction

Neuroblastoma (NB) is the most common extracranial solid tumor diagnosed in childhood, accounting for approximately 8% of all pediatric cancers and 15% of childhood cancer mortality [1]. This heterogeneous disease presents a wide range of biological and clinical manifestations, ranging from spontaneous regression to widespread and highly aggressive, chemo-resistant tumors. Several genetic and epigenetic alterations have been described in NB cells, including amplification of the oncogene *MYCN*, mutations of *ALK* and segmental chromosomal alterations [2,3]. Current therapy for high-risk (HR) NB, accounting for 55–60% of patients, includes intensive induction of chemotherapy followed by surgery; consolidation with high-dose multi-agent chemotherapy, autologous stem cell transplantation and radiation therapy; treatment of minimal residual disease using differentiation agents and immunotherapy. Despite intensive multimodal therapy, long-term survival in HR-NB is less than 50% [4].

In 2009, our group described the calcium-sensing receptor (CaSR) as a tumor suppressor gene involved in the differentiation pathways of NB [5,6]. CaSR is a class C G-protein coupled receptor (GPCR), mainly expressed in parathyroid glands, responsible for the maintenance of calcium (Ca^2+^) homeostasis. High plasma Ca^2+^ concentrations activate CaSR, leading to a suppression of parathyroid hormone (PTH) secretion, reduction in Ca^2+^ excretion by the kidneys, and Ca^2+^ deposition in the bones [7]. This GPCR is also expressed in different tissues, such as the intestine, lungs, brain, pancreas, cardiac and immune system, where it was shown to play diverse roles in cellular processes, including proliferation, differentiation, gene expression and apoptosis [7]. Furthermore, it has been shown that different GPCR ligands are able to stabilize alternative receptor structures, thus, triggering different physiological responses with potential tissue-selective effects [8]. The development of drugs modulating the CaSR function made the receptor an attractive therapeutic target to explore. 

We previously reported that targeting CaSR with its positive allosteric modulator cinacalcet (CIN) induced endoplasmic reticulum (ER)-stress-coupled apoptosis and cell differentiation in NB cell lines. More importantly, CIN delayed in vivo NB tumor growth and increased the expression of genes involved in cell differentiation [9]. However, these encouraging results came with an associated drawback, since CaSR activation by CIN resulted in reduced plasma Ca^2+^ levels, which were unmanageable in patients [10,11].

Considering the biased signaling properties of CaSR, we aimed at identifying and characterizing other positive allosteric modulators of CaSR that might exert NB-specific effects, without inducing clinically relevant hypocalcemia. We hereby describe a structurally different calcimimetic, AC-265347, as an effective therapeutic agent for NB, since it reduces NB tumor growth by promoting cytodifferentiation while maintaining plasma calcium levels.

## 2. Results

### 2.1. AC-265347 Inhibits NB Tumor Growth While Keeping Normal Plasma Calcium Levels 

**Figure 1:**
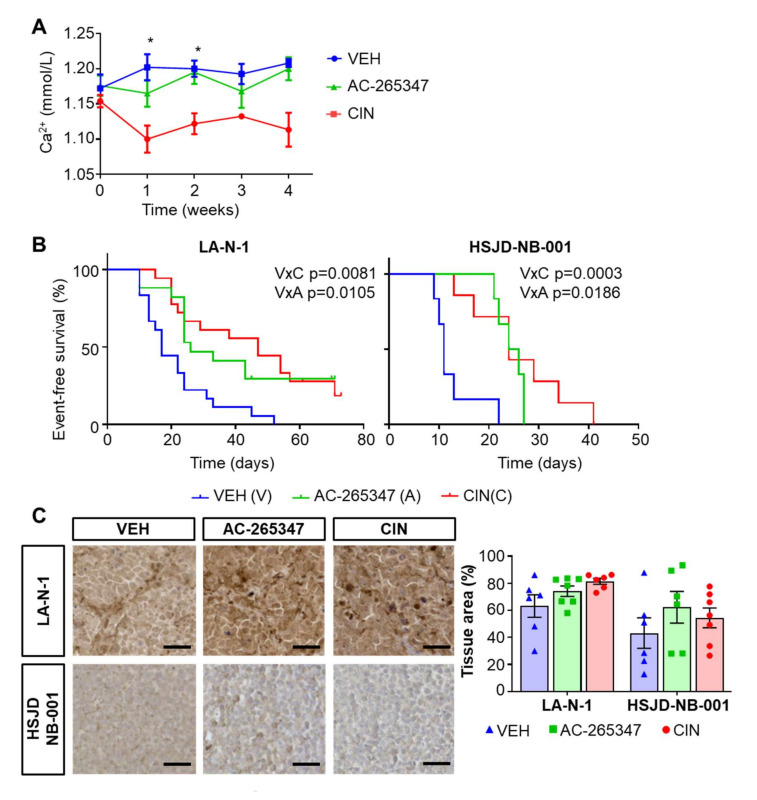
AC-265347 delays NB tumor growth while maintaining plasma calcium levels. (**A**) Healthy athymic mice were treated with 10 mg/kg of AC-265347, CIN or their vehicle (*n* = 5). Blood samples were collected from tail vein and ionised plasma calcium concentration (mmol/L) was measured weekly. Each point represents mean ± SEM of each group. Statistical significance was calculated using One-way ANOVA with Dunn’s correction. * *p* < 0.005. (**B**) Event-free survival (EFS) rates of mice bearing LA-N-1 xenografts (*n* = 17) or HSJD-NB-001 (*n* = 6) treated with 10 mg/kg of AC-265347, CIN or their vehicle. Log-rank statistics with a Bonferroni correction test was used to compare statistical significance between groups (LA-N-1: *p* = 0.0186, VEH vs. AC-265347; *p* = 0.0003, VEH vs. CIN; *p* = 1, CIN vs. AC-265347; HSJ-NB-001: *p* = 0.0105, VEH vs. AC-265347; *p* = 0.0081, VEH vs. CIN; *p* = 1, CIN vs. AC-265347). (**C**) Left: Formalin-fixed, paraffin-embedded sections of LA-N-1 and HSJD-NB-001 tumors exposed to treatment were stained with anti-CaSR. Right: Images were acquired with TissueFAXS at ×20 magnification and percentage of positive area per tumor was quantified using STRATA Quest. Statistical analysis between groups was calculated using a Mann–Whitney test (*p* > 0.05 for all the comparisons). Each symbol represents an individual tumor and bars represent mean ± SEM. Scale represents 100 µm.

In order to avoid the hypocalcemia induced by CIN, we assessed the effects of a third generation calcimimetic, AC-265347, on a healthy immunocompromised mouse model. Plasma Ca^2+^ concentration levels were determined over four weeks. AC-265347 did not reduce the plasma Ca^2+^ levels when compared to vehicle-treated mice. One-way ANOVA with Dunn’s correction was used to compare statistical significance between groups (*p* < 0.005) (Figure 1A). On the other hand, CIN induced non-symptomatic hypocalcemia, as previously documented [9]. No other toxicities were observed (data not shown).

To explore the anti-tumorigenic effects of AC-265347, we evaluated tumor growth inhibition induced by calcimimetics in three different mouse models, carrying LA-N-1 cells, HSJD-NB-001 or HSJD-NB-004 patient-derived xenografts (PDX) models, with different basal expression levels of CaSR, evaluated in early-passage tissues (p2) (Supplementary Figure S1A). AC-265347 delayed NB tumor growth, similar to CIN, in two in vivo models, LA-N-1 and HSJD-NB-001 (Figure 1B). Log-rank statistics with a Bonferroni correction test were used to compare statistical significance between groups (LA-N-1: *p* = 0.0186, VEH vs. AC-265347; *p* = 0.0003, VEH vs. CIN; *p* = 1, CIN vs. AC-265347; HSJD-NB-001: *p* = 0.0105, VEH vs. AC-265347; *p* = 0.0081, VEH vs. CIN; *p* = 1, CIN vs. AC-265347). A similar tendency was observed in HSJD-NB-004 tumor-growth delay (Supplementary Figure S1B). 

Next, we explored the effect of AC-265347 on CaSR expression levels in the eight tumor samples exposed the longest time to each drug, since we previously observed that CIN-responding tumors were those most exposed to this calcimimetic [9]. Immunohistochemistry revealed an upregulation in CaSR expression induced by AC-265347, only in HSJD-NB-001. As described, CIN exposure resulted in an increase in CaSR in both mouse models. However, this upregulation was not significantly different. Statistical analysis between groups was calculated using a Mann–Whitney test (*p* > 0.05 for all the comparisons) (Figure 1C).

### 2.2. Expression of NB Differentiation Markers Is Increased by Exposure to Calcimimetics

**Figure 2:**
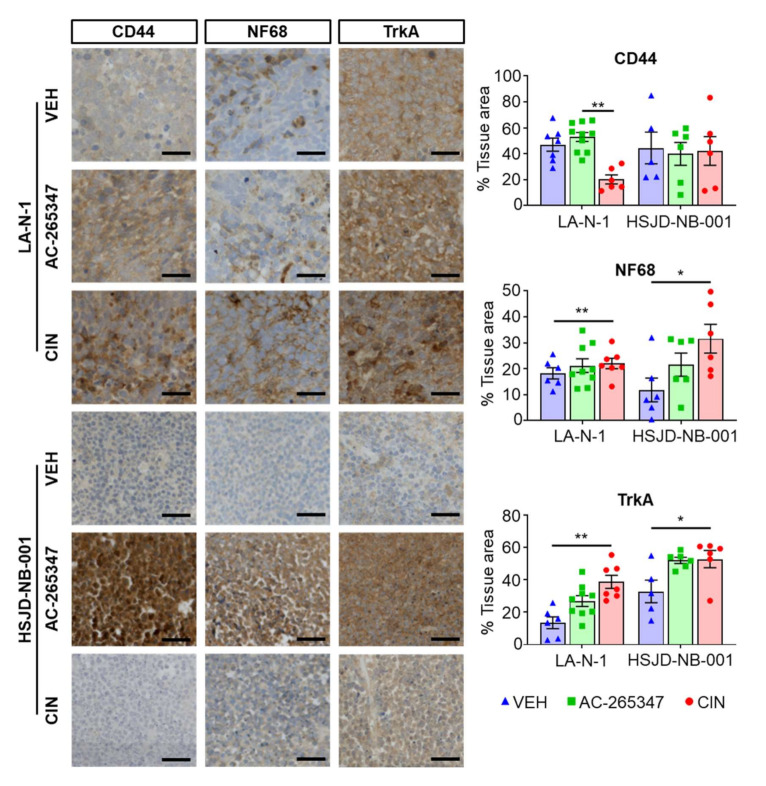
Expression of NB differentiation markers is increased by exposure to calcimimetics. (**Left**) Immunohistochemistry performed with anti-CD44, anti-NF68 and anti-TrkA antibodies in sections of LA-N-1 and HSJD-NB-001 tumors exposed to calcimimetics or vehicle. (**Right**) Whole tumor scan was acquired with TissueFAXS at ×20 magnification and percentage of positive area per tumor was quantified using STRATA Quest. Each symbol represents an individual tumor and lines represent mean ± SD. Significance was calculated using One-way ANOVE using a Dunn’s correction test. * *p* < 0.05, ** *p* < 0.01. Scale represents 100 µm.

As both drugs delayed tumor growth, we decided to evaluate several histological cell proliferation and differentiation markers in the tumors exposed for the longest time to both calcimimetics. No differences were observed in cell proliferation in either model, by quantification of the specific cell proliferation marker Ki67 (data not shown). Expression levels of NF68 and TrkA, two well-described neural differentiation markers, increased by AC-265347 treatment, as observed when mice were treated with CIN. However, only CIN induced downregulation of CD44 expression, an adhesion protein highly expressed in undifferentiated, multipotent neural crest-like NB cells (Figure 2).

### 2.3. AC-265347 and CIN Induce a Different Gene Expression Pattern in NB Xenograft Models 

To explore the effects induced by AC-265347, we evaluated the mRNA expression levels by RT-qPCR of the eight tumors exposed for the longest period of time to calcimimetics, relative to the vehicle-treated tumors (Table 1). Increased expression of *CaSR* was observed in both mouse models exposed to AC-265347, similar to that induced by CIN. This fold increase was exacerbated in HSJD-NB-001 tumors. As seen in Table 1, even though the differentiation effect induced by both calcimimetics was similar, as we observed an upregulation of *NEFL*, *NTRK1*, *NTRK3*, *S100B* and *TUBB3*, we could appreciate a different gene expression pattern induced by AC-265347. No upregulation of several transcripts involved in Ca^2+^ signaling and ER stress were observed in the two NB xenograft models exposed to AC-265347. Moreover, an increase in *CHD5*, a well-recognized tumor suppressor gene in NB, was induced only by CIN. In keeping with differentiation induction, expression levels of *MYCN* and *TGFβ* were significantly modified only by AC-265347. However, these effects were model dependent. Interestingly, AC-265347 exposure also resulted in an increase in cancer testis antigens (CTAs) in both animal models, since an upregulation of *CTCFL*, *GAGE* family, *NY-ESO-1* and *SSX4/4B* was observed (Table 1).

### 2.4. NB Cell Lines Respond Differently to Chronic Exposure of AC-265347

**Figure 3:**
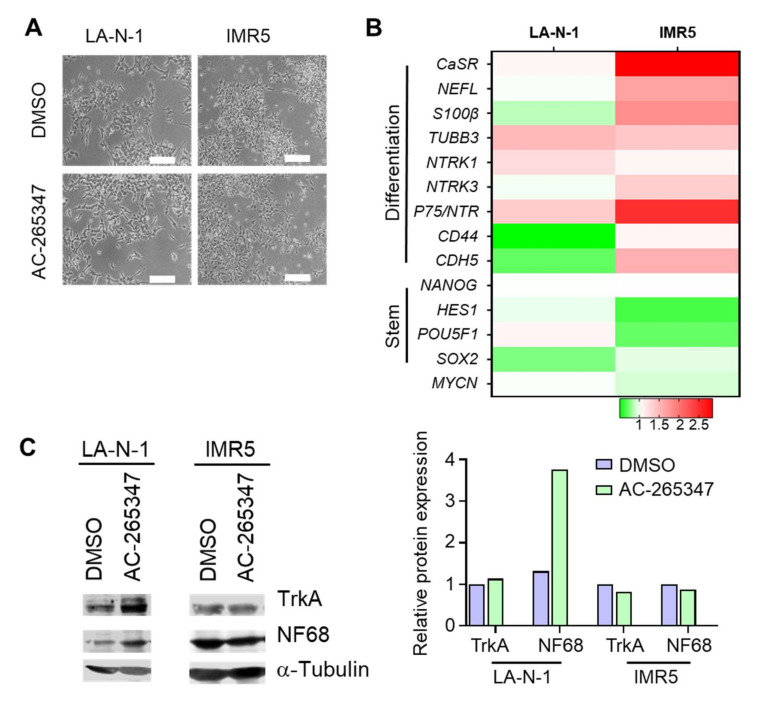
NB cell lines respond differently to chronic exposure to AC-265347. (**A**) Microphotography of LA-N-1 and IMR5 cells treated for 14 days with 1µM of AC-265347 or their vehicle (DMSO). Scale bar represents 500 px. (**B**) Heat-map comparing gene expression patterns in NB cells treated for 14 days with 1 µM of AC-265347. Three independent experiments were performed. Statistical significance was calculated using one-way ANOVA with Dunn’s correction test. (**C**) Left: Immunoblot of TrkA, NF68 and α-Tubulin of LA-N-1 and IMR5 cells exposed to AC265347 for 14 days. Right: Quantification of immunoblots was done using ImageJ and bars represent band intensity relative to α-Tubulin.

To explore the effects on cell differentiation induced by AC-265347, we decided to analyze LA-N-1 and IMR5 NB cell lines, after a long exposure to this calcimimetic. The calculation of the AC-265347 half maximal inhibitory concentration (IC_50_), of six NB cell lines, showed elevated IC_50_ values, making us reason that similar to CIN, AC-265347 does not exert a cytotoxic effect on NB cell lines (Supplementary Figure S2). Exposure of 14 days to a concentration lower than IC_50_ resulted in morphological features of neuronal differentiation in both cell lines, being more pronounced in IMR5 cells showing a more complex dendrite network and flatter colonies (Figure 3A). In keeping with differentiation induction, a higher upregulation of *NEFL*, *S100B*, *NTRK3* and *p75/NTR* was observed in IMR5 cells. Notably, a downregulation of different genes associated with a stem-like phenotype, such as *NANOG*, *HES1*, *POU5F1* and *SOX2*, was observed in both cell lines, as evaluated by RT-qPCR (Figure 3B). Moreover, protein levels of NF68 were upregulated in LA-N-1 cells exposed to AC-265347 (Figure 3C).

### 2.5. Acute Exposure to Calcimimetics Differently Affects NB Cells

**Figure 4:**
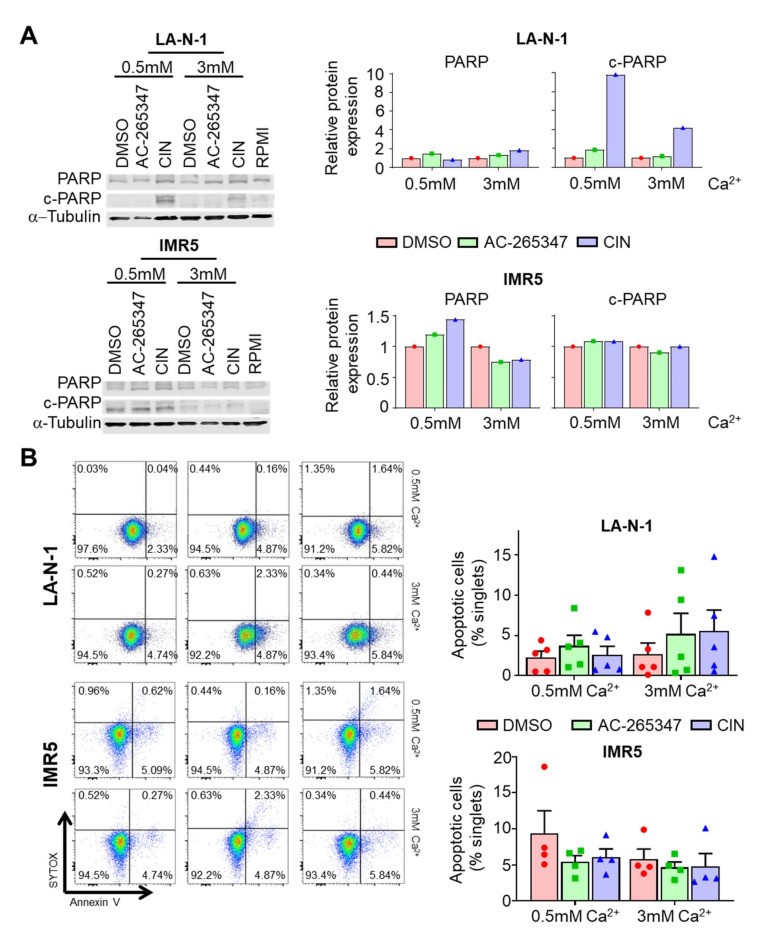
Acute exposure to AC-265347 differently affects NB cells. (**A**) Left: Immunoblot of cleaved-Parp (c-Parp) and full-length PARP (PARP) from LA-N-1 and IMR5 cells exposed to 1µM of AC-265347, CIN or vehicle (DMSO) in the presence of 0.5 or 3 mM of CaCl_2_, for 24 h. Right: Quantification of immunoblots; bars represent band intensity relative to α-tubulin. (**B**) Left: IMR5 and LA-N-1 cells were stained with SYTOX and Annexin V after a 16-h exposure to 1µM of CIN, AC-265347 or their vehicle (DMSO) in the presence of 0.5 or 3 mM of CaCl_2_. Right: Annexin V positive cells in each treatment were quantified by flow cytometry. Statistical analysis between groups was calculated using a Mann–Whitney test (*p* > 0.05 for all the comparisons). Each symbol represents an individual experiment and bar represents mean + SEM.

To gain further insight into the signaling pathways promoted by CaSR in NB cells after AC-265347 exposure, we treated an in vitro model, widely used to characterize signaling pathways activated by this GPCR, in the presence of 0.5 or 3 mM CaCl_2_ [6]. As expected, CIN prompted cleavage of PARP when LA-N-1 cells were exposed to 0.5 or 3 mM of CaCl_2_ (9.8- and 4.1-fold, respectively), even though this effect was cell line dependent. However, we observed no significant changes on the full-length PARP protein when either LA-N-1 or IMR5 cells were exposed to AC-265347 (Figure 4A).

Analyses by flow cytometry of annexin V-SYTOX-stained cells after exposure to calcimimetics, in the presence of either low or high Ca^2+^ concentration, confirmed that exposure to AC-265347 did not result in cell death by apoptosis (Figure 4B).

### 2.6. Genome-Wide Analyses of CIN and AC-265347-Treated Neuroblastoma Xenografts Reveal Biased Signaling 

**Figure 5:**
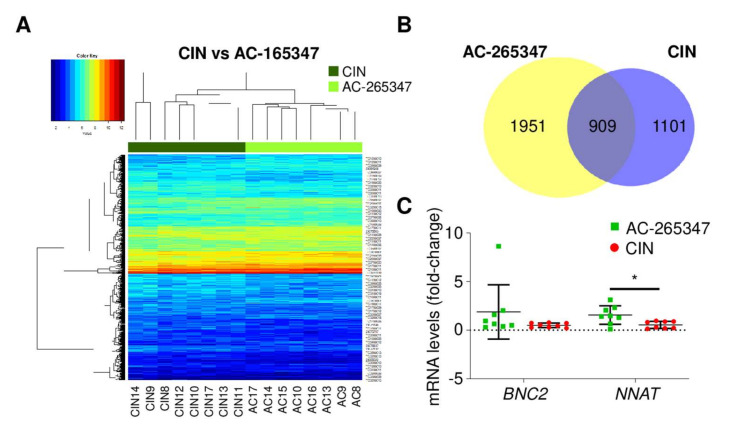

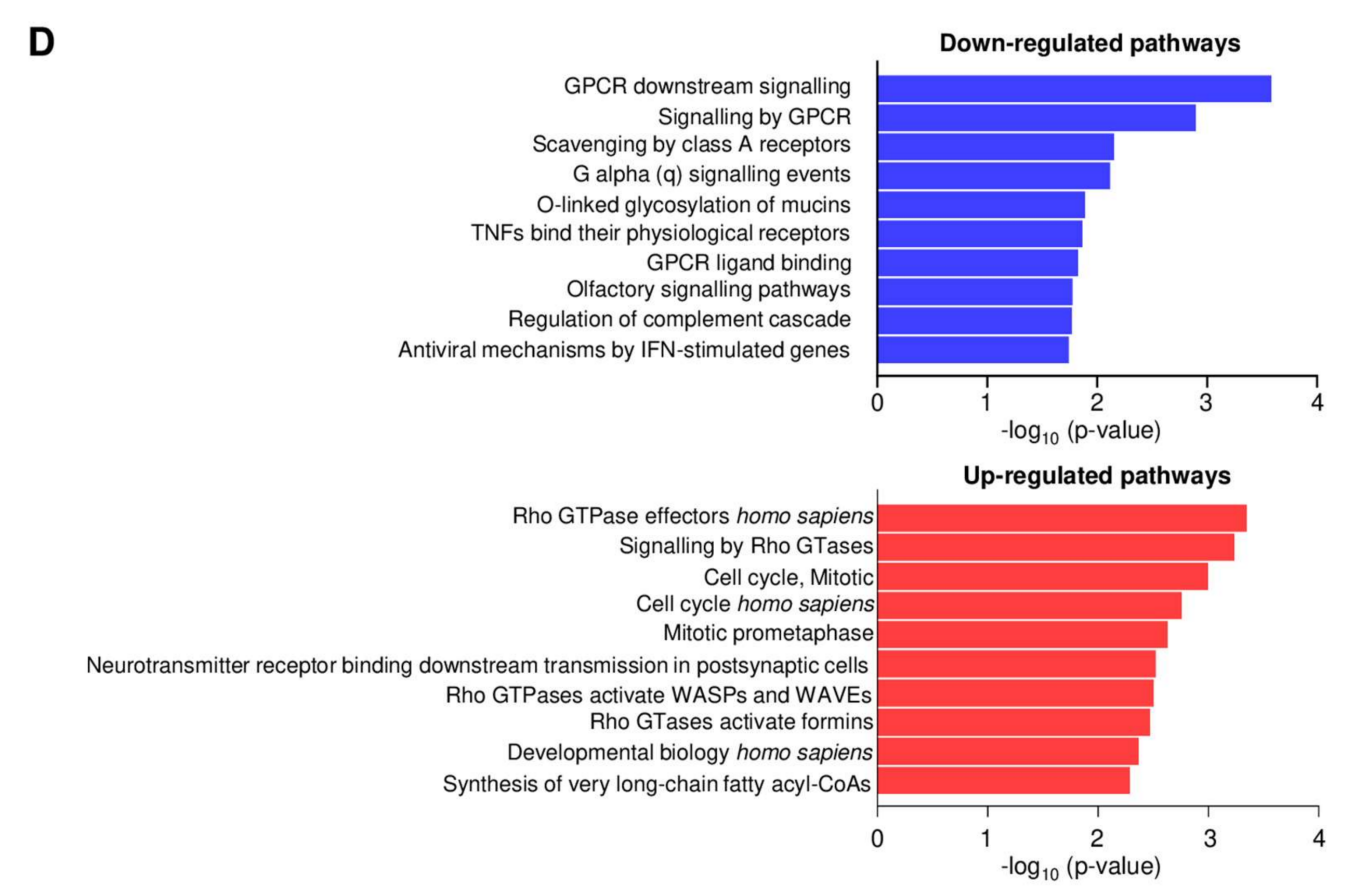
Genome-wide expression analyses of AC-265447 and CIN-treated neuroblastoma xenografts reveal biased signaling by both calcimimetics. (**A**) Hierarchical clustering comparing gene expression pattern in LA-N-1 xenografts exposed to AC-265347 or CIN. These were the eight tumors exposed for the longest time to the drugs. (**B**) Venn diagrams for AC-265347 and CIN differential expressed genes in LA-N-1 xenografts exposed to these calcimimetics. Each diagram contains the number of genes represented. Common genes are represented in dark shading. (**C**) Relative expression of *NNAT* and *BNC2* mRNA in AC-265347 and CIN-treated LA-N-1 tumors. Statistical significance was calculated using two-tailed Mann–Whitney *U* test to compare all AC-265347 and CIN xenografts. * *p* < 0.05. (**D**) Most significantly enriched terms in LA-N-1 xenografts exposed to calcimimetics. Blue color indicates downregulated and red upregulated gene sets.

Since our results suggest that both calcimimetics behave different, a genome-wide expression analysis was performed to compare human gene expression profile in AC-265347 and CIN-treated LA-N-1 xenografts. A supervised hierarchical cluster analysis was performed (Figure 5A and Supplementary Figure S3A,B), showing two well differentiated groups (Figure 5A). Low stringency analyses (*p* < 0.05) identified 2860 (AC-265347 vs. VEH) and 2010 (CIN vs. VEH) differentially expressed genes, of which 909 were common (Figure 5B). In this analysis, the cut-off point applied was *p* < 0.05 and absolute fold change >1. This approach identified only two genes that were upregulated by AC-265347 treatment, *Basonuclin-2* (*BNC2*) and *Neuronatin* (*NNAT*). Validation of microarray data and analysis of differentially expressed genes was conducted by RT-qPCR. The analysis included the two genes with the highest level of significance (*BNC2* and *NNAT*) (Figure 5C). A second less restrictive analysis was performed (*p* < 0.05 and absolute fold change >0.35) and identified a gene profile encompassing 772 probes, corresponding to 340 upregulated and 432 downregulated transcripts.

To identify relevant biological processes that might be involved, Reactome enrichment analysis was performed. The most significant upregulated biological pathway induced by AC-265347 relates to RHO GTPases signaling. Among downregulated genes, the most differentially expressed were involved in GPCR downstream signaling (Figure 5D).

## 3. Discussion

Refractory neuroblastoma represents a clinical challenge and novel therapeutic strategies are needed to change the outcome for the patients. Our group previously described that CIN, an allosteric modulator of CaSR, reduces NB tumor growth by promoting differentiation and ER-stress-mediated apoptosis [9]. However, clinical use of CIN is limited because it induces clinically significant hypocalcemia [12], likely resulting from both suppressed renal calcium reabsorption and calcitonin-mediated inhibition of bone resorption via CaSR activation in thyroid C-cells [13].

Different calcimimetics stabilize receptor conformations that regulate CaSR signaling towards distinct intracellular pathways, a phenomenon termed biased modulation [12]. The identification of allosteric modulators that bias CaSR receptors provides an opportunity to develop new drugs with desirable physiological responses. Hence, we sought to identify a new calcimimetic that biased CaSR signaling towards a distinct intracellular pathway, showing tumor suppression properties similar to CIN, without affecting plasma calcium levels. This work identifies a structurally distinct calcimimetic, AC-265347, shown to have a minor effect on plasma Ca^2+^ levels, while keeping a strong effect in vivo inhibiting PTH [14]. Furthermore, AC-265347 presents a promising therapeutic activity against NB.

We showed that exposure to AC-265347 did not significantly reduce plasma Ca^2+^ levels on healthy immunocompromised athymic nude mice, consistent with previously published data [14]. Notably, AC-265347 significantly inhibited tumor growth of LA-N-1 xenograft models, similar to CIN. Surprisingly, when we evaluated the effect of AC-265347 and CIN in two PDX models, only HSJD-NB-001 responded to both calcimimetics, suggesting a model-dependent response. In keeping with our data, high levels of CaSR correlated with sensitivity to calcimimetics. From the two PDX models included in this study, HSJD-NB-004 showed lower CaSR protein expression levels, limiting the effect of the calcimimetics. In both responsive animal models, tumor-growth inhibition came along with an upregulation of CaSR expression, corroborating previously published data, demonstrating that calcimimetics modulate both the expression and activity of CaSR [9,15,16]. This data would be clinically relevant, since most unfavorable NBs exhibit low levels of CaSR and increased expression is associated with differentiated, favorable, neuroblastic tumors [5].

Our first analyses, based on our previous findings, showed that AC-265347 exposure resulted in a more differentiated phenotype in both responsive animal models, similar to CIN. Quite unexpectedly, although the differentiation effect induced by both calcimimetics was similar, we could appreciate a different expression pattern of specific cell differentiation markers. While both calcimimetics induced upregulation of TrkA and NF68 in both animal models, downregulation of CD44 protein levels was observed only in CIN-exposed mice. High levels of TrkA and NF68 are associated with favorable outcome [17,18,19,20]. There have been controversies about the significance of CD44 expression in NB, and its relationship with tumor progression. However, most recently, a new report showed high expression of CD44 to be associated with lower survival, independent of MYCN amplification [21]. Accordingly, analysis of gene expression levels corroborated the differentiation effect, since upregulation of *S100B* and *TUBB3* was observed, showing a marked effect on CIN-treated tumors. S100*β* is involved in cell proliferation, survival and differentiation [22]. In NB, patients with S100*β* protein-positive cells had a more favorable outcome [23]. Expression of *CHD5*, the neuron-specific marker of outcome in NB [24,25], was increased only by CIN exposure. The different gene expression pattern associated to both calcimimetics suggests the activation of different signaling cascades. Alternatively, these results could be explained by distinct differentiation markers increasing at different times of the differentiation process [26]. Interestingly, in both responsive animal models, a moderate downregulation of *MYCN* was induced. High expression of *MYCN* correlates with high-risk disease and poor prognosis. Accordingly, differentiation of NB cells is associated with *MYCN* reduced expression [27,28]. Downregulation of *MYCN* was also reported in NB cells undergoing differentiation upon exposure to retinoids [29]. The differentiation effects of sustained in vitro exposure to calcimimetics reproduced, quite notably, the effects induced by these drugs in vivo, making clear that CIN and AC-265347 exerted their anti-tumorigenic activities via different mechanisms.

Apoptosis caused by endoplasmic reticulum stress can be induced by the accumulation of unfolded proteins, resulting from disrupted Ca^2+^-dependent chaperone function in the ER [30,31]. According to our previous reports, CIN triggered ER stress in LA-N-1 and HSJD-NB-001 xenograft models by up-regulating *RYR2*, the ryanodine receptor 2. RyR dysfunction shapes ER Ca^2+^ dynamics in pancreatic *β* cells and regulates both the unfolded protein response (UPR) activation and cell death [9,32]. As expected, ER stress was not induced by AC-265347. AC-265347 preferentially modulates pERK1/2 and IP1 accumulation over Ca^2+^ mobilization [12]. Moreover, AC-265347 may have a reduced propensity to cross cell membranes to pharmaco-chaperone misfolded receptors trapped in the ER and Golgi compartment, as previously described [12]. Since a decrease in tumor growth was observed, differentiation is suggested as the main effect of AC-265347 in NB tumors.

CTAs are a large family of tumor-associated antigens, considered ideal targets for immunotherapy, based on their tumor-restricted expression pattern, immunogenicity and putative role in oncogenesis [33]. AC-265347, as per our previously published data on the effect of CIN in the LA-N-1 xenograft models, significantly increased CTAs gene expression. Immunotherapies targeting specific CTAs have reached clinical development. A phase 1 study used an autologous CTA-specific dendritic cell vaccine combined with decitabine for the treatment of HR-NB patients and other pediatric tumors [34]. The effect induced by AC-265347 on CTAs expression could prime for CTA-based immunotherapies. On the other hand, in the LA-N-1 mouse model, a statistically significant *TGFB1* increase was induced by AC-265347. TGF*β* is a cytokine involved in both suppressive and inflammatory immune responses [35]. Recently it has been described that activation of CaSR induced the expression and secretion of anti-inflammatory cytokines in colon cancer cells overexpressing CaSR [36]. Understanding if calcimimetics modulate the expression of cytokines involved in the immune response against NB might contribute to potentiate future immunotherapy strategies.

Finally, a genome-wide expression analysis of the LA-N-1 mouse model was performed to shed light on the biased signaling and differentiation induction by both calcimimetics. As expected, no differences were observed at a restrictive threshold, suggesting that both treatments differ minimally in their molecular mechanism of action. Only two genes were differentially expressed, *NNAT* and *BNC2*. *NNAT* is an imprinted gene described to be upregulated in the IMR32 NB cell line after treatment with demethylating agents, inducing changes in cell morphology (extension of neural processes), suggesting cell differentiation. Moreover, high expression of NNAT was associated with good prognosis in NB [37]. In contrast, the literature is scarce about BNC2. Published data suggest that BNC2 could be an activator of a subset of interferon-regulated genes and might thereby act as a tumor suppressor gene [38]. Further analysis should be performed to explain the role of NNAT and BNC2 in AC-255347-promoting NB cell differentiation. 

Pathway enrichment analysis, applying less restrictive conditions, showed mechanistic insights. In keeping with our previous results and the published literature, we observed that CIN-enhanced biased calcium signaling pathways [8,9,12]. Conversely, AC-265347 biased to signaling by Rho GTPases. AC-265347, unlike CIN, favored the ERK1/2 phosphorylation pathway that regulates a wide variety of cellular processes, including cell proliferation, migration and differentiation [39]. Phosphorylation of ERK1/2 is key in the process of neurite outgrowth of neurons [40]. Moreover, cell differentiation induced by retinoic acid is driven by the activation of RhoA and the downstream MAPK signaling pathways [41]. Our results suggest that AC-265347 could have a differential effect on ERK1/2 phosphorylation and RhoA activation, explaining the anti-tumorigenic potential of this calcimimetic. Further studies will be necessary to establish whether RhoA activation is involved in the AC-265347 differentiation effect in NB. 

Finally, AC-265347 and CIN activate different Gα-protein subunits, leading to activation of different signaling cascades in HEK293 cells overexpressing CaSR [42]. Our results clearly showed that activation of CaSR by both calcimimetics induced different signaling cascades, suggesting different anti-tumorigenic pathways in NB. Future studies should focus on understanding the molecular mechanisms behind the anti-tumorigenic effect of AC-265347 coupled to Gα-protein in this cellular context.

Taken together, this study highlights the importance of biased allosteric modulation when targeting GPCRs in cancer. We identified a new calcimimetic with anti-tumoral properties, without causing clinically significant hypocalcemia. The capacity of this calcimimetic to promote the differentiation of malignant NB cells provides new opportunities, alone or in combination with other drugs, for the treatment of high-risk NB.

## 4. Material and Methods

### 4.1. Cell Lines

Neuroblastoma cell lines LA-N-1, IMR5, SH-SY5Y, SK-N-JD and SK-N-LP were obtained from the repository at the Institut de Recerca Hospital Sant Joan de Déu (Barcelona, Spain). Cells were grown in Roswell Park Memorial Institute (RPMI)-1640 medium supplemented with 10% fetal bovine serum (Invitrogen, Carlsbad, CA, USA), 2 mM L-glutamine, penicillin (100 U/mL) and streptomycin (100 μg/mL), at 37 °C and 5%. Mycoplasma polymerase chain reaction (PCR) tests were performed monthly. Characterization of neuroblastoma cell lines included analysis of *MYCN* amplification [6], *TP53* sequence and authentication by STR profiles. 

### 4.2. Reagents

Cinacalcet (CIN) was obtained from Selleckchem (Houston, TX, USA). Stock solutions were prepared in DMSO and stored at −80 °C. For in vivo studies, CIN was prepared from Mimpara^®®^ tablets (Amgen, CA, USA), homogenized using a mortar and reconstituted as described [9]. AC-265347 was obtained from Sigma-Aldrich (St Louis, MO, USA) and stock solutions were prepared in DMSO. For animal studies AC-265347 was reconstituted with 5% DMSO and 90% PEG400 (Sigma-Aldrich) to a final concentration of 20 mg/mL. 

### 4.3. Cell Viability Assays

NB cells (1000–2500) were seeded into 96-well plates, six replicate wells for each line and condition. Concentrations ranging from 200 μM to 0.78 μM of AC-265347 or CIN were added 24 h after seeding. IC_50_ (drug concentrations achieving 50% decrease in cell viability) was quantified with CellTiter 96^®®^ Aqueous Non-Radioactive Cell Proliferation Assay (Promega, WI, USA) following the manufacturer’s protocol at indicated times. Absorbance measured at 490 nm using a TECAN INFINITE FNano + (Männedorf, Switzerland) was directly proportional to the number of living cells in each well. The IC_50_ was calculated at 72 h using GraphPad Prism software, version 7.01 for windows (GraphPad Software, San Diego, CA, USA).

### 4.4. Apoptosis Assays

LA-N-1 and IMR5 (5 × 10^5^) cells were cultured in 6-well plates and treated with either 1 μM of AC-265347 or CIN and allowed to grow for 48 h. Then, cells were collected and resuspended in 50 μL of Annexin Ca^2+^ buffer containing 5% Pacific Blue Annexin V and 1% SYTOX^®®^ Green (Invitrogen) and incubated at RT for 20 min. Next, cells were washed with 200 μL of Annexin V buffer. Cell acquisition was performed in Novocyte Flow cytometer and analyzed with the NovoExpress (ACEA Biosciences, San Diego, CA, USA). Analysis of flow cytometry data was performed using FlowJo (Becton, Dickinson & Company, Franklin Lakes, NJ, USA). 

### 4.5. RNA Extraction and Quantitative RT-PCR

Cell cultures were harvested and total RNA was extracted using TRI Reagent (Invitrogen) according to the manufacturer’s instructions. Total RNA (1 μg) was reverse transcribed and qPCRs were carried out as previously described [5,6]. Real-time PCR runs were performed in a 7500 SDS system using gene-specific Assays on Demand and Taqman Universal PCR Master Mix, or specific primers (Supplementary Table S1) and SYBRGreen (Applied Biosystems, Forster City, CA, USA). Relative expression levels were calculated as previously described [9].

### 4.6. Immunoblots

Cells were harvested and cell pellets resuspended in radioimmunoprecipitation (RIPA) buffer (10 mM Tris-HCl pH 6.8, 1 mM EDTA, 150 mM NaCl, 1% SDS (all Sigma-Aldrich)) complemented with a protease inhibitors cocktail (Roche, Basel, Switzerland). Proteins (50–75 µg) were electrophoresed in 8–15% SDS-PAGE and transferred onto nitrocellulose membranes. Primary antibodies (TrKA (Santa Cruz, CA, USA), NF68 (Thermofisher, MA, USA), CaSR and PARP (Abcam, Cambridge, UK), cleaved-PARP (Cell Signaling Technologies, Danvers, MA, USA) and alpha-Tubulin (Sigma-Aldrich)), were incubated overnight, followed by secondary antibody incubation IRDye680RD goat anti-mouse IgG (Li-COR #926-68070) or IRDye800CW goat anti-rabbit IgG (Li-COR#926-32211). Blots were visualized and quantified using Li-COR Odyssey system (Li-COR Biosciences, Lincoln, NE, USA).

### 4.7. Histology and Immunohistochemistry (IHC)

Sections from formalin-fixed, paraffin-embedded tissues (4 μm) were stained with hematoxylin-eosin (H&E) or processed for immunohistochemistry to analyze Ki67, CaSR, CD44, TrkA or NF68 protein expression as described [9]. Stained slides were scanned using an Axio Imager Z2 (Zeiss) attached to a tissue cytometer TissueFAXS (TissueGnostics, Vienna, Austria) using an EC-Plan Neofluar 20×/0.5 objective. Acquisition of the whole tumor area was done automatically with the acquisition of 5 × 5 mm fields of view (FOVs). Stitching between each FOV was done automatically. Quantification of IHC was done using StrataQuest software (TissueGnostics) by detecting positive DAG signal. Briefly, percentage of positively stained areas was recorded, as an alternative to individual cells. After color segmentation (separation of blue and brown signals), the image was converted to black and white to improve differentiation of color intensities. To avoid recording unspecific binding of antibodies to necrotic areas, the algorithm differentiates between high-, medium- and low-intensity signals (the intensity criteria were defined by the user for each IHC antibody). Medium- and low-intensity areas were used to quantify the positive signal for each tumor. Percentage of positive signal was calculated as follows: (“Medium-intensity area” + “Low-intensity area”)/“Total tissue area”. 

### 4.8. Mouse Xenograft Models and Calcemia Study

Four to six-week female Athymic nude-Fox1 nu/nu mice (Charles River Wilmigton, MA, USA) were subcutaneously injected with 7.5 × 10^6^ LA-N-1 cells resuspended in PBS: Matrigel (Becton Dickinson, Flanklin Lakes, NJ, USA) in both flanks of the mouse. Low-passage (p2) PDX models HSJD-NB-001 and HSJD-NB-004, both *MYCN-A* and *TP53* mutated, were generated as previously described [43]. Tumor size was measured using a caliper three times per week. Tumor volume (V) was calculated with the formula: V = (L × W^2^)/2, where L is the length and W is the width of a xenograft. When tumors reached a volume of 175 mm^3^ animals were randomized in three groups to receive CIN, AC-265347 or its vehicle by oral gavage, 5 days per week. Tumors were excised when volume reached 1500 mm^3^. For calcemia study, healthy mice were treated and blood samples were collected from tail vein 4 h after drug administration. Total Ca^2+^ levels were measured once per week using an EPOC reader (Alere Healthcare, MA, USA). For PDX generation, written informed consent was obtained from patients/parents/legal guardians and procedures were approved by the Institutional Review Boards. All animal experiments were carried out under procedures approved by the Institutional Animal Research Ethics Committee and followed European Guidelines (EU directive 2010/63/EU). 

### 4.9. Microarray Processing and Analysis

Total RNA was extracted from 8 tumor xenografts exposed to CIN, AC-265347 or their vehicle. Genome-wide expression analysis was performed using Affymetrix Clarion^TM^ S Assay for human samples (Affymetrix, Santa Cruz, CA, USA) following manufacturer’s protocol. Microarrays data were analyzed as previously described [9]. Differentially expressed genes (*p* value ≤ 0.05 and LogFC ≥ 1) obtained from comparing eight tumors of each treatment group were used for a gene enrichment analysis against the Gene Ontology using Enrichr [44,45]. A less restrictive gene set (*p* value ≤ 0.05 and LogFC ≥ 0.35) was also conducted using Enrichr analysis. Microarray data have been deposited in the Gene Expression Omnibus database (Accession number GSE195577).

### 4.10. Statistical Analysis

All statistical analysis was done using GraphPad Prism, version 7.01 for windows (GraphPad Software) Statistical analysis between two groups was calculated using a Mann–Whitney test, a one-way ANOVA with a Dunn’s correction test was used when three or more groups were compared. EFS survival curves were compared using Log-rank statistics with a Bonferroni correction test for multiple comparisons, significance was considered when *p* < 0.05. Gene expression analysis and IHC quantifications from mouse xenograft models were compared using one-way ANOVA with a Dunn’s post-correction test for multiple comparisons.

## Figures and Tables

**Table 1:** Gene expression analyses of neuroblastoma xenografts following AC-265347 and CIN treatment conducted by RT-qPCR.

		LA-N-1		HSJD-NB001	
		AC-265347	CIN	AC-265347	CIN
Differentiation	*CASR*	2.51	2.21	17.33	15.46
	*NEFL*	1.47	2.31	1.23	1.98
	*NTRK1*	1.76	3.67	0.69	0.9
	*NTRK2*	1.25	1.81	1.12	0.95
	*S100B*	1.31	1.51	1.23	1.98
	*TUBB3*	1.41	5.24	0.94	0.82
	*CHD5*	1.1	11.91	1.5	1.07
	*MYCN*	1	0.93	0.68	0.9
Ca^2+^ signaling	*TGFB1*	1.56	1.44	0.44	0.92
	*RYR*	1.15	2.26	1.16	1.75
ER-stress	*CHOP*	0.78	1.58	1.18	1.14
	*BID*	0.91	2.15	1.31	0.87
CTAs	*CTCFL*	1.9	2.47	1.22	1
	*GAGE12J*	3.56	9.9	0.32	0.13
	*MAGEA2*	1.01	0.98	0.92	0.45
	*MAGEA3*	1.16	2.86	3.5	0.49
	*MAGEA6*	1.4	2.48	2.36	0.43
	*NY-ESO-1*	1.68	5.24	1.32	0.43
	*SSX4/4B*	3.05	10.64	2.76	0.69
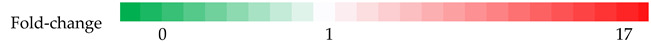					

## Data Availability

Not applicable.

## Supplementary Materials

The following supporting information can be downloaded at: https://www.mdpi.com/article/10.3390/ijms23084323/s1.
