# Supplementary material for: AC-265347 Inhibits Neuroblastoma Tumor Growth by Induction of Differentiation without Causing Hypocalcemia
Source: Int J Mol Sci. 2022 Apr 13;23(8):4323. doi: 10.3390/ijms23084323 (PMC9027928; doi:10.3390/ijms23084323)
Supplement: Supplementary file 1 [file ijms-23-04323-s001.zip › ijms-1680068-supplementary.pdf]

International Journal of
Molecular Sciences

MDPI

Supplementary Materials

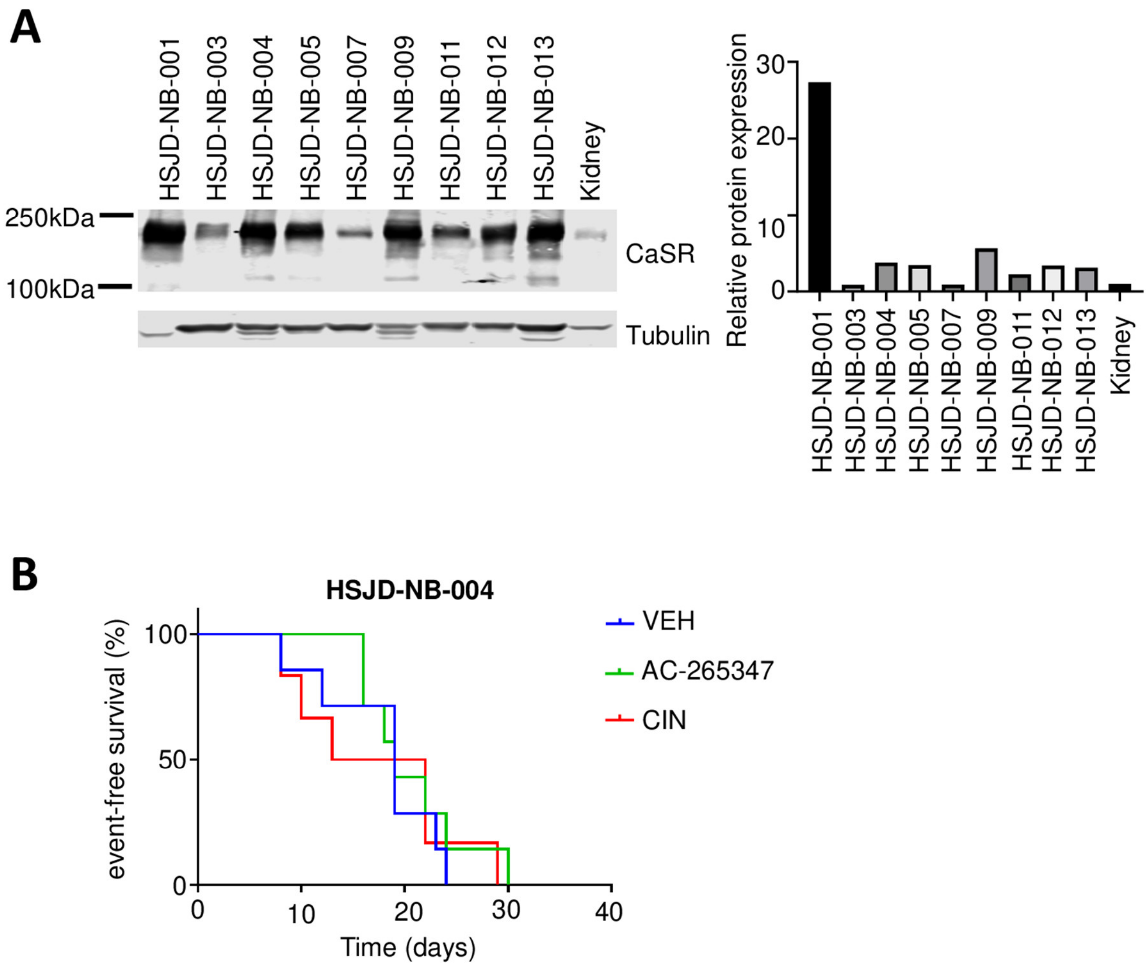

Figure S1. CaSR protein expression levels limits the effect of the calcimimetics. **A–** Right: Immunoblot showing CaSR expression levels of NB PDX models generated by our group. Left: Bands intensity was quantified relative to α-tubulin. **B–** EFS curves of mice bearing HSJD-NB-004 exposed to 10mg/kg of AC-265347, CIN or their vehicle (n=8). Log-rank statistics with Bonferroni's correction test was used to compare statistical significances between treatment groups (p=1 for all comparisons).

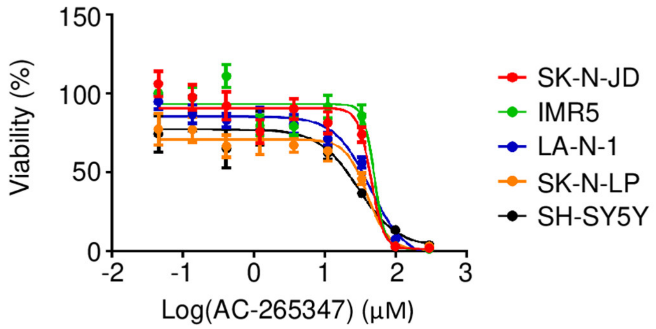

Figure S2. AC-265347 does not exert a cytotoxic effect on NB cell lines. Indicated neuroblastoma cell lines were treated with consecutive lower concentration of AC-265347 (starting at 200 μM) to evaluate cytotoxic activity. Each point represents mean ± SEM of six technical replicates and IC_{50} was calculated using GraphPad.

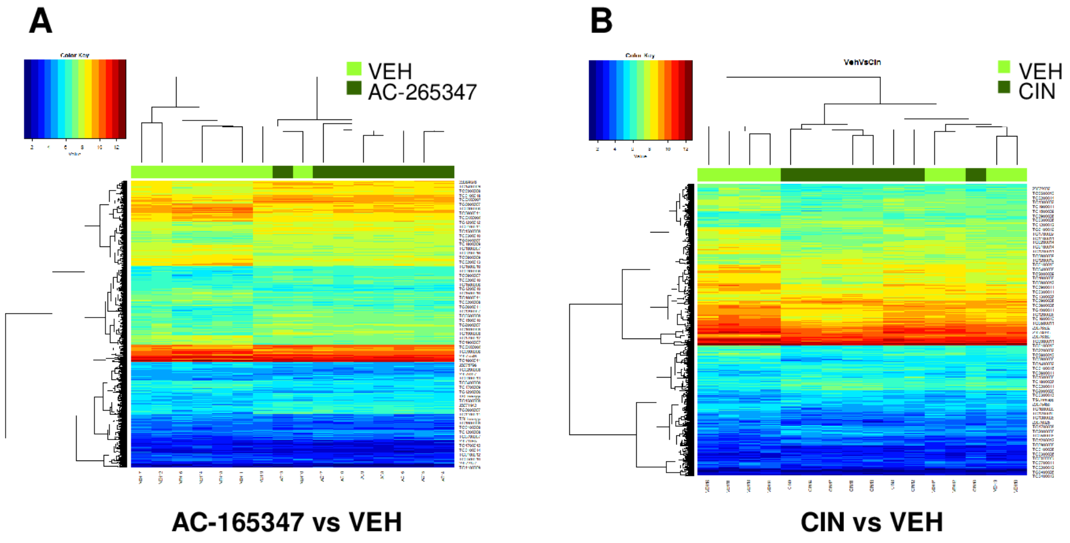

Figure S3. Genome-wide expression analyses of AC-265347, CIN and Vehicle treated neuroblastoma xenografts. Hierarchical clustering of gene expression pattern in LA-N-1 xenografts models exposed to **A**– AC-265347 and **B**– CIN compared to Vehicle treated mice. These were the eight tumors exposed for the longest time to the drugs.

Table S1. Primers and Taqman probes used for genes expression analysis.

Gene	Assays on Demand	Forward	Reverse
BID	-	CTTAGCCAGAAATGGGATG	AGTCACAGCTATCTTCCAG
CASR	Hs01047795_m1	-	-
CD44	Hs01075861_m1	-	-
CDH5	-	CCACAACGAGGGCATCATCA	GAGGTCGATGGTGGGGTCTG
CHOP	Hs99999172_m1	-	-
CTCFL	-	GCACGGAAAAGCGACCTACG	TGAGGGCATAGCGTTCATGG
GAGE-P1	-	GCAGCTGCTCAGGAGGGAGA	TGTGGGTGACCCTGTTCCTG
GAGE-P2	-	GGCCGAAGCCTGAAGCTCAT	ATTTGGCGGGTCCATCTCCT
GAGE-P3	-	GAGGGAGCATCTGCAGGTCAA	CCTGCCCATCAGGACCATCT
HES1	-		
MAGE-A2	-	AACCAGGCAGTGAGGCCTTG	AGCCTGTCCCCCTCAGAACC

MAGE-A3	-	AAGGTGGCCGAGTTGGTTCA	ACTGCCAATTTCCGACGACA
MAGE-A6	-	CGGGGATCCCAAGAAGCTG	TTTCAATGAGGGCCCTTGGA
MYCN	Hs00232074_m1	-	-
NANOG	-		
NEFL	Hs00196245_m1	-	-
NTRK1	Hs01021011_m1	GACGCTTCGTGTTCAGCTCGCG	CTCGGTTGAGCGTTCTTGC
NTRK3	Hs00176797_m1		
NY-ESO-1	-	TGCTTGAGTTCTACCTCGCCAT	GCTCCTGCGGGCCAG
POU5F1	-		
p75/NTR	Hs00609977_m1	-	
RYR	-	GCTCAAGGTGGTGGTCATCG	TTGAAGACCGGGAGGTGGAC
S100-β	Hs00902901_m1	-	
SOX2	-		-
SSX4/4B	-	CACCCTCCCACCTTTCATGC	GGACGTTCAACCTGATTCCTGTG
TBP	-	GAACATCATGGATCAGAACAACAG	ATTGGTGTTCTGAATAGGCTGTG
TGFβ1	-	CTGGAGAGGGCCCAGCATCT	CGCACGCAGCAGTTCTTCTC
TUBB3	Hs00801390_m1	-	-
